# Anthrax: has the clinical milieu changed since 2001?

**DOI:** 10.3402/jchimp.v2i2.18191

**Published:** 2012-07-16

**Authors:** Amesh A. Adalja

**Affiliations:** 1 Center for Biosecurity of UPMC, Baltimore, MD, USA; 2 Department of Critical Care Medicine, Department of Emergency Medicine, Department of Medicine Division of Infectious Diseases, University of Pittsburgh Medical Center, Pittsburgh, PA, USA

**Keywords:** Anthrax, bioterrorism

## Abstract

Since the anthrax attacks of 2001 (Amerithrax), several important improvements in the knowledge of *Bacillus anthracis* and the clinical condition it causes have occurred. While much remains to be known about the optimal management of anthrax patients, several approaches that were not widely utilized, available, or known in 2001 would be used in the treatment of critically ill anthrax patients in 2012.

Since the anthrax attacks of 2001 (Amerithrax), several important improvements in the knowledge of *Bacillus anthracis* and the clinical condition it causes have occurred. While much remains to be known about the optimal management of anthrax patients, several approaches that were not widely utilized, available, or known in 2001 would be used in the treatment of critically ill anthrax patients in 2012.

## General improvement in critical care will benefit anthrax patients

In the last decade, the care delivered in intensive care units (ICUs) – the primary treatment site for inhalational anthrax patients (which may become scarce in a mass casualty setting) – has undergone a paradigm shift. Specifically, improvements in the treatment of two conditions that would directly impact the care of anthrax patients: Acute Lung Injury (ALI)/Acute Respiratory Distress Syndrome (ARDS) and severe sepsis/septic shock have occurred.

## ALI/ARDS

While anthrax does not cause a strict pneumonic process, the physiological derangements caused by infection with *B. anthracis* can lead to ALI and ARDS with diffuse damage to the alveoli of the lungs culminating in mechanical ventilator support– an intervention 50% of the Amerithrax inhalational victims received (1). As ALI/ARDS is known to complicate up to 50% of severely septic patients (2), irrespective of cause, it is not surprising that among the six (of 11) Amerithrax inhalational anthrax victims in whom the data is available, two-thirds had evidence of ALI/ARDS at presentation (3). Utilizing appropriate ventilator settings that do not exacerbate lung damage is crucial in the management of these patients. The key element to this approach is the use of low tidal volume ventilation (6 ml/kg), spurred by the publication of the results of the ARDSNet trial in 2000, and now widely practiced in intensive care units worldwide. This use of low tidal volume mechanical ventilation can potentially decrease the mortality of ALI/ARDS patients by 9% (4). It is unclear whether inhalational anthrax patients, who have a propensity for large pleural effusions, would benefit to the degree seen with standard ARDS cases. Additionally, in severe ARDS, rescue therapies such as extra-corporeal membrane oxygenation (ECMO) and high frequency oscillatory ventilation (HFOV) have also been implemented with some success in severely hypoxic patients (5). However, it remains to be seen whether ECMO, with its usual need for systemic anticoagulation, would be feasible in inhalational anthrax patients who exhibit hemorrhagic manifestations.

## Severe sepsis/septic shock

The treatment of severe sepsis/septic shock has been revolutionized by the concept of early goal directed therapy (EGDT) which aims to restore the physiologic derangements inherent in these conditions to near normal, including targeting a mixed oxygen venous oxygen saturation of 70% or greater. The seminal trial by Rivers and colleagues, published in November of 2001, which demonstrated a 16% reduction in mortality, sparked the development of a protocol-based approach to the resuscitation and care of the severe sepsis/septic shock patient exemplified by the Surviving Sepsis Campaign and treatment guidelines (released in 2004, revised in 2008) (6, 7). Of the 2001 inhalational Amerithrax patients, the majority met criteria for severe sepsis and/or septic shock, meriting EGDT. Other interventions advocated, and now routinely initiated, for patients in severe sepsis and septic shock include: sedation/paralysis protocols; stress ulcer prophylaxis; deep venous thrombosis (DVT) prophylaxis; and glucose control protocols (7). Lastly, in patients with severe sepsis or septic shock, one meta-analysis assessing the effect of the administration of polyclonal intravenous immunoglobulin (IVIG) revealed a 26% survival benefit (8).

## Routine drainage of anthrax-related pleural effusions

The management of one specific anthrax complication has also been further defined since 2001. The development of pleural effusions is a well described complication of inhalational anthrax and was exhibited by all 11 Amerithrax inhalation patients. The drainage of anthrax-related pleural effusions has now been advocated as a key intervention to improve survival. In a systematic review of inhalational anthrax cases from 1900–2005, pleural fluid drainage was found to be highly statistically correlated with survival. In addition to the expansion in lung volume that occurs, the high level of anthrax toxins present in the pleural fluid contributes to the ongoing illness and removing the nidus of toxins facilitates recovery (1, 9).

## Antimicrobial therapy armamentarium expansion

The antimicrobial treatment of anthrax largely remains the same as it was in 2001. To date, the FDA has approved four antimicrobial agents for the treatment and/or prevention of anthrax. Penicillin, doxycycline, and ciprofloxacin were approved for this indication pre-2001; levofloxacin was approved for this indication in 2004 (10). However, many other available antimicrobial agents are effective against strains of *B. anthracis* and FDA approval is not needed for this specific indication as these agents can and are used ‘off-label’. Since the Amerithrax attacks, five new antimicrobial agents with activity against *B. anthracis* have been FDA-approved (for other indications): ertapenem, doripenem, tigecycline, telavancin, gemifloxacin, telithromycin, and daptomycin (11–16).

## Diagnostic testing: Culture remains the mainstay

Routine bacteriologic culture of *B. anthracis* from body fluids remains the chief means of diagnosing anthrax today, as it was in 2001. Polymerase chain reaction (PCR)-based methods may also be applied in research/reference laboratory settings. In 2004, however, the FDA cleared a qualitative IgG and IgM serologic test (QuickELISA Anthrax-PA Kit) for detection of antibodies directed against *B. anthracis* in serum which will augment diagnostic capabilities, post-exposure screening activities, and can also be utilized to demonstrate seroconversion after vaccination (9).

## Vaccination schedule abbreviated; administration route changed

In 2001, the pre-exposure administration of the anthrax vaccine (AVA) had required a series of subcutaneous injections administered t 0, 2, and 4 weeks and 6, 12, and 18 months with annual boosters. High rates of adverse reactions at the injection site, prompted study of alternative schedules. In 2008, the FDA modified the administration schedule reducing the number of injections to 5 (eliminating the 2 weeks injection) and changing the route of administration to intramuscular—an alteration that did not result in any decrement in immunogenicity (9).

The three dose subcutaneous regimen (0, 2, and 4 weeks) of AVA utilized as post-exposure prophylaxis (PEP) remains, as it was in 2001, an FDA unapproved – but recommended—intervention requiring administration as an Investigational New Drug (IND) (9).

No 2nd generation vaccines have received FDA approval to date.

## No new immunotherapeutic approaches widely available

Although much research has been expended in the development of additional immunologic countermeasures including protective antigen (PA) targeted monoclonal and ordinary antibodies – both included in the Strategic National Stockpile (SNS) – none have been FDA approved yet. The use of antibody based therapy, while considered novel today, is not a new phenomenon as its efficacy has been known for over a century (9). In a systematic review of inhalational anthrax cases from 1900–2005, the use of serum therapy was statistically correlated with survival (1). Moreover, in a non-fatal 2006 inhalational anthrax case related to an African drum exposure, anthrax immune globulin (AIG) was administered as an IND (8) and has since been used in several sporadic cases that have occurred worldwide. Immunologic therapeutics would have a role in augmenting antimicrobial therapy and would be a major countermeasure to be utilized against multi-drug resistant strains of *B. anthracis*.

## Future developments that would augment our ability to treat anthrax post-2001

Since the attacks of 2001, several anthrax cases have occurred in a variety of circumstances including: musicians exposed to animal skin drums harboring *B. anthracis* spores, injection drug users self-infusing heroin contaminated with *B. anthracis*, and a singer contracting the first reported gastrointestinal anthrax case in the US after performing with an African drumming troupe. Each of these cases provided an opportunity to trial new approaches to anthrax utilizing the full prowess of critical care and infectious disease physicians coupled with the knowledge gained during 2001. Indeed, the mortality rate (45%) of inhalational anthrax patients in 2001 was substantially lower than historical controls (92%) attesting to the improvement in care of these critically ill patients; in 2011, a further decrement in mortality could be expected with full implementation of sepsis guideline compliant therapy. With full guidelines compliance, the Surviving Sepsis Campaign 165 center study demonstrating an absolute 2-year mortality reduction of nearly 8 percent (17).

Irrespective of the improvements in the care of the critically ill, the prevention and treatment of future cases of anthrax – both natural and unnatural – could be greatly enhanced with the availability and development of specific countermeasures (and related policies) which include:

1. A 2nd generation vaccine that does not require repeated administrations for efficacy
2. The availability and FDA approval of AIG and PA-targeted monoclonal antibodies
3. The FDA approval of the AVA vaccine as a routine part of PEP
4. The widespread availability (and FDA approval) of PCR based assays to detect *B. anthracis* in body fluids (blood, cerebrospinal fluid, ascites, pleural fluid, etc.)
5. The maintenance of adequate supplies of the materials needed to drain pleural effusions in national stockpiles of medical equipment
